# Associations of GSTM1*0 and GSTA1*A genotypes with the risk of cardiovascular death among hemodialyses patients

**DOI:** 10.1186/1471-2369-15-12

**Published:** 2014-01-14

**Authors:** Sonja Suvakov, Tatjana Damjanovic, Tatjana Pekmezovic, Jovana Jakovljevic, Ana Savic-Radojevic, Marija Pljesa-Ercegovac, Slavica Radovanovic, Dragan V Simic, Steva Pljesa, Milos Zarkovic, Jasmina Mimic-Oka, Nada Dimkovic, Tatjana Simic

**Affiliations:** 1 Institute of Medical and Clinical Biochemistry, Faculty of Medicine, University of Belgrade, Pasterova 2, 11000, Belgrade, Serbia; 2 Clinical Department for Renal Diseases, Zvezdara University Medical Center, Belgrade, Serbia; 3 Institute of Epidemiology, Faculty of Medicine, University of Belgrade, Belgrade, Serbia; 4 Institute of Medical Physiology, Faculty of Medicine, University of Belgrade, Belgrade, Serbia; 5 Department of Cardiology, Medical Center “Bezanijska Kosa”, Belgrade, Serbia; 6 Clinic for Cardiovascular Diseases, Clinical Centre of Serbia, Belgrade, Serbia; 7 Department of Nephrology and Hemodialysis, University Teaching Hospital Zemun, Belgrade, Serbia; 8 Clinic of Endocrinology, Clinical Center of Serbia, Belgrade, Serbia; 9 Faculty of Medicine, University of Belgrade, Belgrade, Serbia

**Keywords:** Cardiovascular disease, ESRD, Polymorphism, Mortality risk, Oxidative stress

## Abstract

**Background:**

The presence of glutathione transferase (GST) M1 null genotype (*GSTM1*-null) in end-stage renal disease (ESRD) patients is associated with lower overall survival rate in comparison to those with *GSTM1*-active variants. We examined association between *GSTM1* and *GSTT1* deletion polymorphisms as well as SNPs in *GSTA1/*rs3957357 and *GSTP1*/rs1695 genes with overall and cause-specific cardiovascular mortality in ESRD patients.

**Methods:**

Total of 199 patients undergoing hemodialysis were included in the study. Median value of time elapsed from dialysis initiation until the death, or the end of follow-up was 8 ± 5 years. The effect of *GSTM1, GSTT1, GSTP1* and *GSTA1* gene polymorphisms on predicting overall and specific cardiovascular outcomes (myocardial infarction, MI or stroke) was analyzed using Cox regression model, and differences in survival were determined by Kaplan-Meier.

**Results:**

*GSTM1*-null genotype in ESRD patients was found to be independent predictor of overall and cardiovascular mortality. However, after false discovery rate and Bonferroni corrections this effect was lost. The borderline effect modification by wild-type GSTA1*A/*A genotype on associations between *GSTM1*-null and analyzed outcomes was found only for death from stroke. Homozygous carriers of combined GSTM1*0/GSTA1*A genotype exhibited significantly shorter time to death of stroke or MI in comparison with carriers of either *GSTM1*-active or at least one GSTA1*B gene variant. The best survival rate regarding cardiovascular outcome was found for ESRD patients with combined *GSTM1*-active and mutant GSTA1*B/*B genotype.

**Conclusions:**

Combined GSTM1*0/GSTA1*A genotypes might be considered as genetic markers for cardiovascular death risk in ESRD patients, which may permit targeting of preventive and early intervention.

## Background

Oxidative stress in end stage renal disease (ESRD) patients is considered to be the cornerstone of atherosclerotic process. Carotid artery intima-media thickness in chronic hemodialysis patients correlates with lipid peroxidation byproducts [1], while serum malondialdehyde (MDA) is a strong predictor of prevalent cardiovascular disease in these patients [2]. Very recently it has been shown that susceptibility to oxidative stress in ESRD is influenced by the genetic polymorphism in antioxidant and detoxifying enzymes glutathione transferases (GST). The family of cytosolic GSTs comprises different classes including the Alpha (GSTA), Mu (GSTM), Pi (GSTP) and Theta (GSTT) class. Approximately half of the general population lacks GSTM1 enzyme activity, due to a homozygous deletion of the *GSTM1* gene [3]. In the case of GSTT1, gene homozygous deletion, present in about 20% of Caucasians, leads to the lack of GSTT1 enzyme activity [4]. Single-nucleotide polymorphism (SNP), resulting in amino acid substitution from isoleucine (Ile) to valine (Val) [5], changes catalytic activity of the GSTP1 enzyme [6]. *GSTA1* polymorphism is represented by three, apparently linked, SNPs which result in differential expression with lower transcriptional activation of the variant GSTA1*B (-567G, -69T, -52A) than common GSTA1*A allele (-567T, -69C,-52G) [7]. According to the presence of various *GST* gene variants in combination, ESRD patients may be stratified in level of oxidative, carbonyl and nitrosative stress.

Since oxidative stress parameters correlate with cardiovascular complications and mortality [8-11], interaction between the uremic state and particular GST genotype would represent a potential mechanism explaining the inter-individual differences in terms of cardiovascular outcome in these patients. In the non-ESRD population, individuals with *GSTM1* and/or *GSTT1-null* genotypes seem to be at higher risk of CVD [12,13]. The observed link between GST polymorphism and CVD was further strengthened in smokers lacking *GSTM1* or *GSTT1* genes [14,15]. The *GSTT1-null* genotype and combined GSTT1*0/GSTM1*0 might be potential determinants of susceptibility to advanced atherosclerosis in patients with type 2 diabetes mellitus [16]. In ESRD patients, only polymorphic expression of *GSTM1* was studied with respect to prognostic significance. Although the presence of *GSTM1-null* genotype in ESRD patients was associated with lower overall survival compared to those with *GSTM1-active* gene variants, specific association of this and other common GST polymorphisms, with cause-specific cardiovascular mortality still has to be addressed. This study examined the association between the deletion polymorphisms in *GSTM1* and *GSTT1* as well as SNPs in *GSTA1* (rs3957357) and *GSTP1* (rs1695) genes with overall and cardiovascular mortality as well as the death from myocardial infarction (MI) and stroke (CVI) in 199 dialysis patients.

## Methods

### Study subjects

A total of 199 patients (84 male and 115 female, mean age 60.0 ± 12.1 years) undergoing hemodialysis three times a week were included in the study. All patients were stable, aged over 21 and with HD vintage > 3 months before the study.

End stage renal failure was due to a hypertensive nephrosclerosis (93), glomerulonephritis (32), diabetic nephropathy (25), polycystic renal disease (19), pyelonephritis (19), Balkan endemic nephropathy (7) and obstructive nephropathy in 4 patients. Patients were treated with single-use dialyzers equipped with low flux and high flux polysulphone membranes (surface area of 1.3-2.1.m^2^). Study protocol was approved by the Belgrade University Faculty of Medicine Ethic Committee and the research was carried out in compliance with the Helsinki Declaration. All the participants provided written informed consent.

During 36 months cardiovascular mortality and all-cause mortality were prospectively registered. Beginning of the study is defined as time when patient started chronic hemodialysis therapy. Information regarding death and causes of death were obtained from hospital records and other relevant documents. Causes of death were classified as cardiovascular death if myocardial infarction and/or stroke occurred. Myocardial infarction was diagnosed by cardiologist on the basis of clinical presentation, ECG parameters and dynamic of enzyme activities. Stroke was diagnosed by neurologist according to clinical presentation and CT scan.

### GST Genotyping

Genomic DNA was isolated from whole blood using the QIAGEN QIAmp kit (Qiagen, Inc., Chatsworth, CA).

*GSTA1 C-69T* polymorphism was determined by polymerase chain reaction–restriction fragment length polymorphism (PCR-RFLP) [17]. Used primers were *GSTA1 C-69T* forward: 5′-TGTTGATTGTTTGCCTGAAATT-3′ and *GSTA1 C-69T* reverse, 5′-GTTAAACGCTGTCACCCGTCCT-3′. Presence of restriction site resulting in two fragments (481bp and 385bp) indicated mutant allele (GSTA1*B/B) and if GSTA1*A/B polymorphism incurred it resulted in one more fragment of 96bp.

*GSTM1* genotyping was performed by multiplex PCR [17]. Used primers were *GSTM1* forward: 5′-GAACTCCCTGAAAAGCTAAAGC-3′ and *GSTM1* reverse: 5′-GTTGGGCTCAAATATACGGTGG-3′. Exon 7 of *CYP1A1* gene was co-amplified and used as an internal control using following primers: *CYP1A1* forward: 5′-GAACTGCCACTTCAGCTGTCT-3′ and *CYP1A1* reverse: 5′-CAGCTGCATTTGGAAGTGCTC-3′. The presence of *GSTM1-*active genotype was detected by the band at 215bp, since the assay does not distinguish heterozygous or homozygous wild type genotypes.

*GSTP1 Ile105Val* polymorphism was analyzed using PCR-RFLP method [17]. Used primers were: *GSTP1 Ile105Val* forward: 5′-ACCCCAGGGCTCTATGGGAA-3′ and *GSTP1 Ile105Val* reverse: 5′-TGAGGGCACAAGAAGCCCCT-3′. Presence of restriction site resulting in two fragments (91bp and 85bp) indicated mutant allele (Val/Val) while if Ile/Val polymorphism incurred it resulted in one more fragment of 176bp.

*GSTT1* genotyping was performed by multiplex PCR [17]. Used primers were *GSTT1*-forward: 5′-TTCCTTACTGGTCCTCACATCTC-3′ and *GSTT1*-reverse: 5′-TCACGGGATCATGGCCAGCA-3′. The assay does not distinguish between heterozygous or homozygous wild type genotypes, therefore the presence of 480bp bands was indicative for *GSTT1-*active genotype.

### Statistical analysis

Survival analysis was performed separately in the total cohort and according to the cardiovascular cause of death. The Kaplan-Meier method was used to estimate the cumulative survival probability. We defined the initiation of dialysis as a zero time and the death as the end-point. The long-rank test was performed for the assessment of differences in survival according to the different categories of variables.

The predictive value of different GST genotypes in overall and cardiovascular mortality was assessed by Cox proportional hazards regression models, adjusted by confounding factors in three models. The numbers of patients included in regression models were the same for the all tested GST polymorphisms. For overall mortality, the number of patients included in Model 1, 2 and 3 were 186, 183 and 169, respectively; for cardiovascular mortality 180, 177 and 166 patients, respectively; for myocardial infarction 189, 186 and 169 patients and for cardiovascular insult 190, 187 and 169 patients, respectively. In Model 1 we adjusted for age and gender. Model 2 included the covariates in Model 1 plus an additional adjustment for the current smoking status. Model 3 included all covariates from Models 1 and 2, plus an additional adjustment for diabetes and cholesterol status. The associations are presented as hazard ratios (HR) with their corresponding 95% confidence intervals (95% CI). To control for multiple comparisons we used multtest package in R. We reported corrected p values obtained by methods that control family-wise error rate [18] and false discovery rate [19,20].

## Results

Table 1 describes the baseline characteristics of the 199 dialysis patients in this study. Time elapsed from dialysis initiation until the death, or the end of follow up, ranged from one to 27 years, with a median value of 8 ± 5 years. There were 55 deaths during the follow-up. The causes of death among our study cohort included cardiovascular diseases (n = 36), infection and sepsis (n = 10), neoplasm (n = 4), gastrointestinal bleeding (n = 2), chronic obstructive lung disease (n = 1), and cachexia (n = 2). Both myocardial infarction and stroke were causes of death in 6 patients. The cause of death was unknown in 2 patients. Although the genotype distribution has been already presented in our previous report [17], herein we place the genotype distribution among ESRD patients in order to better describe of our cohort.

**Table 1:** Baseline characteristics of patients with ESRD

**Variable**	**ESRD patients**
Age (years)^*^	59.1 ± 11.6
Gender, n (%)	
Male	84 (42.2)
Female	115 (57.8)
Diabetes, n (%)	
Present	25 (12.6)
Absent	165 (82.9)
Smoking, n (%)	
Current + Former	54 (27.1)
Never	142 (71.4)
BMI (kg/m^2^)	24.5
Total cholesterol (mmol/L)	4.7 ± 1.1
Urea (mmol/L)	24.4 ± 4.8
Creatinine (μmol/L)	880.2 ± 238.6
Serum iron (μmol/L)	11.3 ± 5.6
***GSTA1***, n (%)	
*A/*A	70 (35.2)
*A/*B	90 (45.2)
*B/*B	39 (19.6)
***GSTM1***, n (%)	
*active*^*a*^	80 (40.2)
*Null*^*b*^	119 (59.8)
***GSTP1***, n (%)	
*Ile/Ile*	75 (37.7)
*Ile/Val*	77 (38.7)
*Val/Val*	47 (23.6)
***GSTT1***, n (%)	
*active*^a^	132 (66.3)
*Null*^b^	67 (33.7)

^a^Active (present) if at least one active allele present. ^b^Inactive (null) if no active alleles present.

^*^x ± SD.

**Figure 1:**
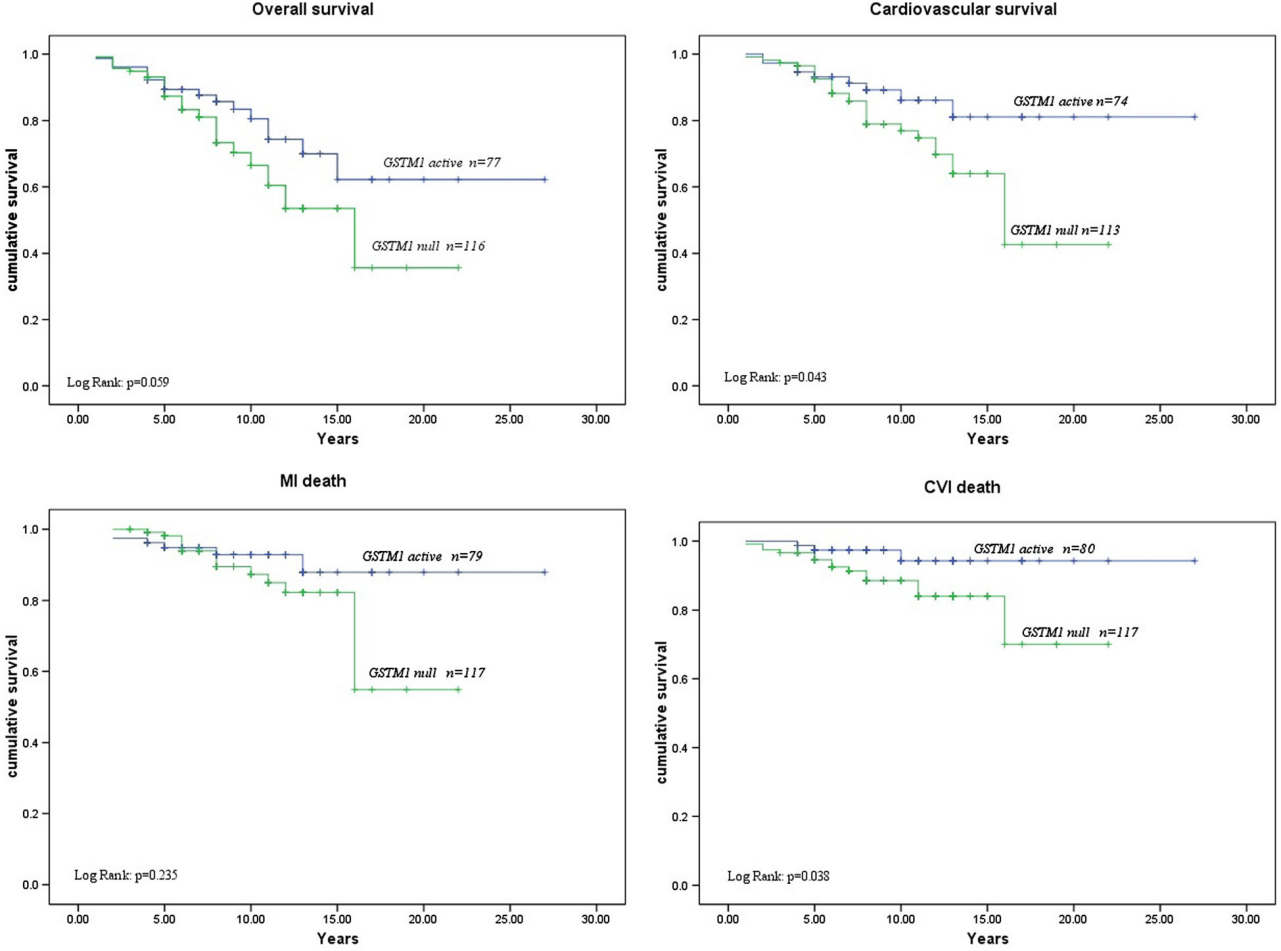
**Kaplan Meier Survival Curves for overall and cardiovascular mortality as well as death of myocardial infarction (MI) and stroke according to *GSTM1 *** **polymorphism.**

Table 2 summarizes the associations between *GSTM1* polymorphism and overall, cardiovascular mortality as well as the death of myocardial infarction and stroke. Multivariable Cox regression analysis was used to determine the independent effect of *GST* gene polymorphisms on predicting these outcomes and included five other variables (age, gender, current smoking status, presence of diabetes, and cholesterol level) in three models (Table 2). The presence of the *GSTM1-null* genotype was an independent predictor of a higher risk for overall and cardiovascular mortality among MHD patients. This genotype had a significant multivariable adjusted (Model 3) HR of 2.08 (CI:1.10-3.92; p = 0.024) and 2.19 (CI:1.00-4.79; p = 0.050) for both overall and cardiovascular mortality, respectively. Moreover, *GSTM1-null* genotype was a strong independent predictor of CVI death with multivariable adjusted (Model 3) HR of 3.80 (CI:1.03-14.02; p = 0.045). However, after multiple testing (Additional file 1: Table S3) none of models remained significant. A Kaplan-Meier survival analysis demonstrated shorter overall (Log Rank: p = 0.059) and cardiovascular survival (Log Rank: p = 0.043) as well as shorter time to death of MI (Log Rank: p = 0.235) or stroke (Log Rank: p = 0.038) after the initiation of dialysis in patients who were homozygous for *GSTM1-null* alleles in comparison with carriers of at least one active *GSTM1* allele (Figure 1).

**Table 2:** ***GSTM1 *** **polymorphism as a predictor for overall and cardiovascular mortality as well as death of myocardial infarction and cerebral vascular insult among 199 ESRD patients after a median follow-up time of 8 yrs by Cox proportional hazards regression models**

**Model 1**^**a**^		**Model 2**^**b**^		**Model 3**^**c**^	
**HR (95% CI)**	**P value**	**HR (95% CI)**	**P value**	**HR (95% CI)**	**P value**
**Risk for overall mortality comparing *GSTM1-null *** **homozygotes to *GSTM1-active *** **carriers**					
1.74 (0.96-3.14)	0.066	1.88 (1.03-3.45)	0.041	2.08 (1.10-3.92)	0.024
**Risk for cardiovascular mortality comparing *GSTM1-null *** **homozygotes to *GSTM- active *** **carriers**					
1.96 (0.95-4.08)	0.070	2.03 (0.97-4.22)	0.060	2.19 (1.01-4.79)	0.050
**Risk for death from myocardial infarction comparing *GSTM1-null *** **homozygotes to *GSTM1-active *** **carriers**					
1.70 (0.65-4.49)	0.281	1.74 (0.66-4.60)	0.263	1.82 (0.63-5.26)	0.270
**Risk for death from CVI comparing *GSTM1-null *** **homozygotes to *GSTM1-active *** **carriers**					
3.38 (0.96-11.94)	0.058	3.54 (0.99-12.56)	0.051	3.80 (1.03-14.02)	0.045

*Abbreviations*: *CI *Confidence Interval, *HR *Hazard Ratio.

^a^Adjusted for age and gender.

^b^Adjusted for the covariates in Model 1 plus an additional adjustment for smoking status.

^c^Adjusted for the covariates in Model 2 plus an additional adjustment for diabetes and cholesterol level.

**Figure 2:**
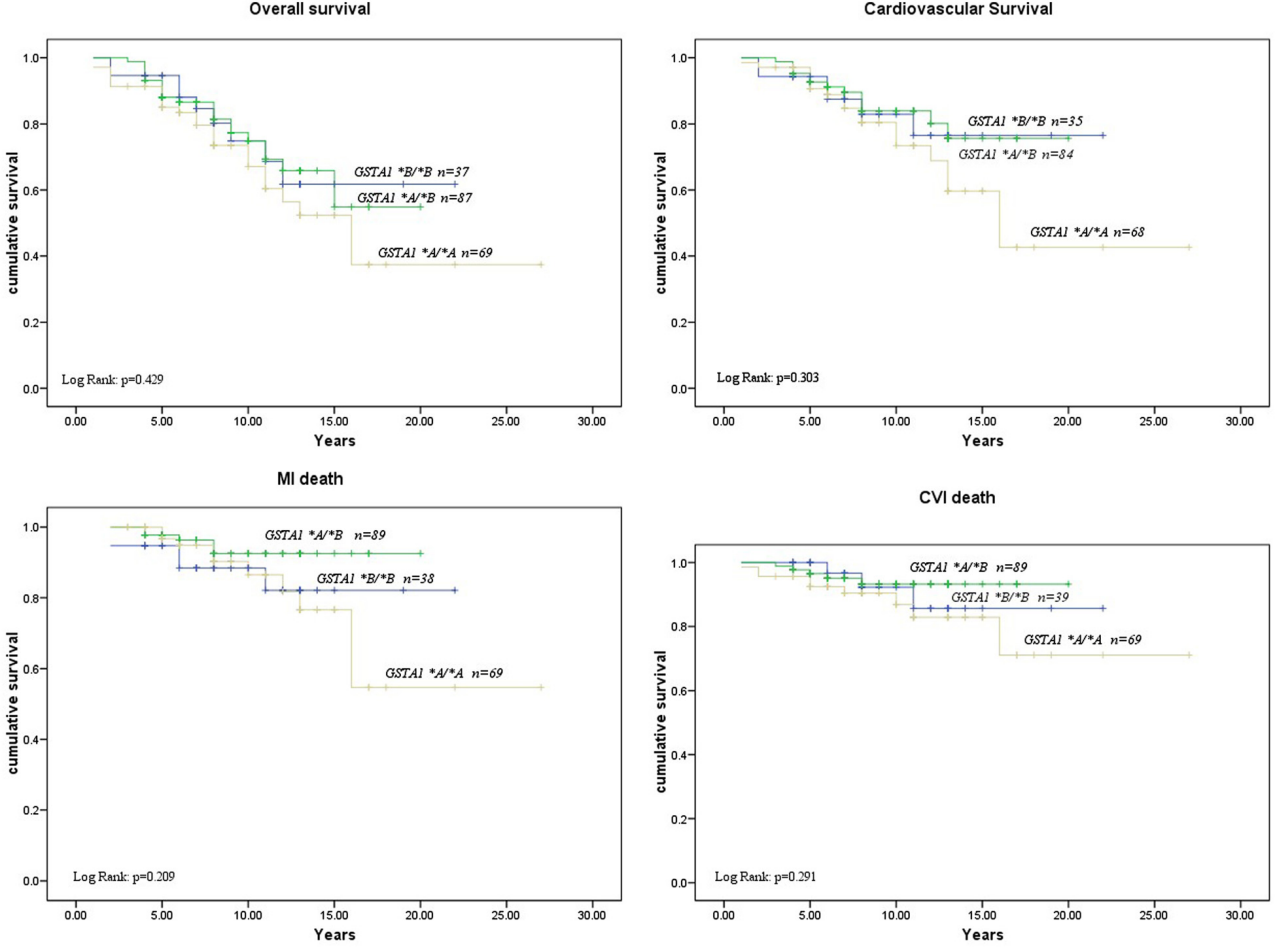
**Kaplan Meier Survival Curves for overall and cardiovascular mortality as well as death of myocardial infarction (MI) and stroke according to *GSTA1 *** **polymorphism.**

Table 3 summarizes the association between *GSTA1* polymorphism with overall and cardiovascular mortality as well as the death of myocardial infarction and stroke. Multivariable adjusted (age, gender, current smoking status, presence of diabetes, and cholesterol level, Model 3) Cox regression analysis has shown the lack of association between *GSTA1* polymorphism and overall mortality (HR = 1.15; 95% CI:0.64-2.07; p = 0.650). However, patients homozygous for GSTA1*A allele demonstrated a non-statistically significant HRs of 1.73, 1.87 and 2.52 for cardiovascular mortality, MI and stroke, respectively. A Kaplan-Meier survival analysis demonstrated shorter, but non-significant overall (Log Rank: p = 0.429) and cardiovascular survival (Log Rank: p = 0.303) as well as shorter time to death of MI (Log Rank: p = 0.209) or stroke (Log Rank: p = 0.291) after the initiation of dialysis in patients who were homozygous for GSTA1*A alleles compared to carriers of at least one GSTA1*B allele (Figure 2).

**Table 3:** ***GSTA1 *** **polymorphism (rs3957357) as a predictor for overall and cardiovascular mortality as well as death of myocardial infarction and cerebral vascular insult among 199 ESRD patients after a median follow-up time of 8 yrs by Cox proportional hazards regression models**

**Model 1**^**a**^		**Model 2**^**b**^		**Model 3**^**c**^	
**HR (95% CI)**	**P value**	**HR (95% CI)**	**P value**	**HR (95% CI)**	**P value**
Risk for overall mortality comparing GSTA1*A homozygotes to GSTA1*B **carriers**					
1.18 (0.68-2.07)	0.556	1.15 (0.65-2.02)	0.635	1.15 (0.64-2.07)	0.650
Risk for cardiovascular mortality comparing GSTA1*A homozygotes to GSTA1*B **carriers**					
1.56 (0.80-3.04)	0.194	1.59 (0.81-3.11)	0.177	1.73 (0.84-3.55)	0.134
Risk for death from myocardial infarction comparing GSTA1*A homozygotes to GSTA1*B **carriers**					
1.70 (0.68-4.29)	0.259	1.72 (0.68-4.36)	0.255	1.87 (0.68-5.15)	0.228
Risk for death from CVI comparing GSTA1*A homozygotes to GSTA1*B **carriers**					
2.25 (0.81-6.21)	0.119	2.32 (0.83-6.46)	0.107	2.52 (0.86-7.36)	0.091

*Abbreviations*: *CI *Confidence Interval, *HR *Hazard Ratio, *CVI* Cerebral Vascular Insult.

^a^Adjusted for age and gender.

^b^Adjusted for the covariates in Model 1 plus an additional adjustment for smoking status.

^c^Adjusted for the covariates in Model 2 plus an additional adjustment for diabetes and cholesterol level.

There were no robust statistical associations between *GSTP1* and *GSTT1* gene variants with overall, cardiovascular mortality as well as the death of myocardial infarction and stroke in dialysis patients according to Cox regression analysis (Additional file 2: Table S1 and Additional file 3: Table S2).

**Figure 3:**
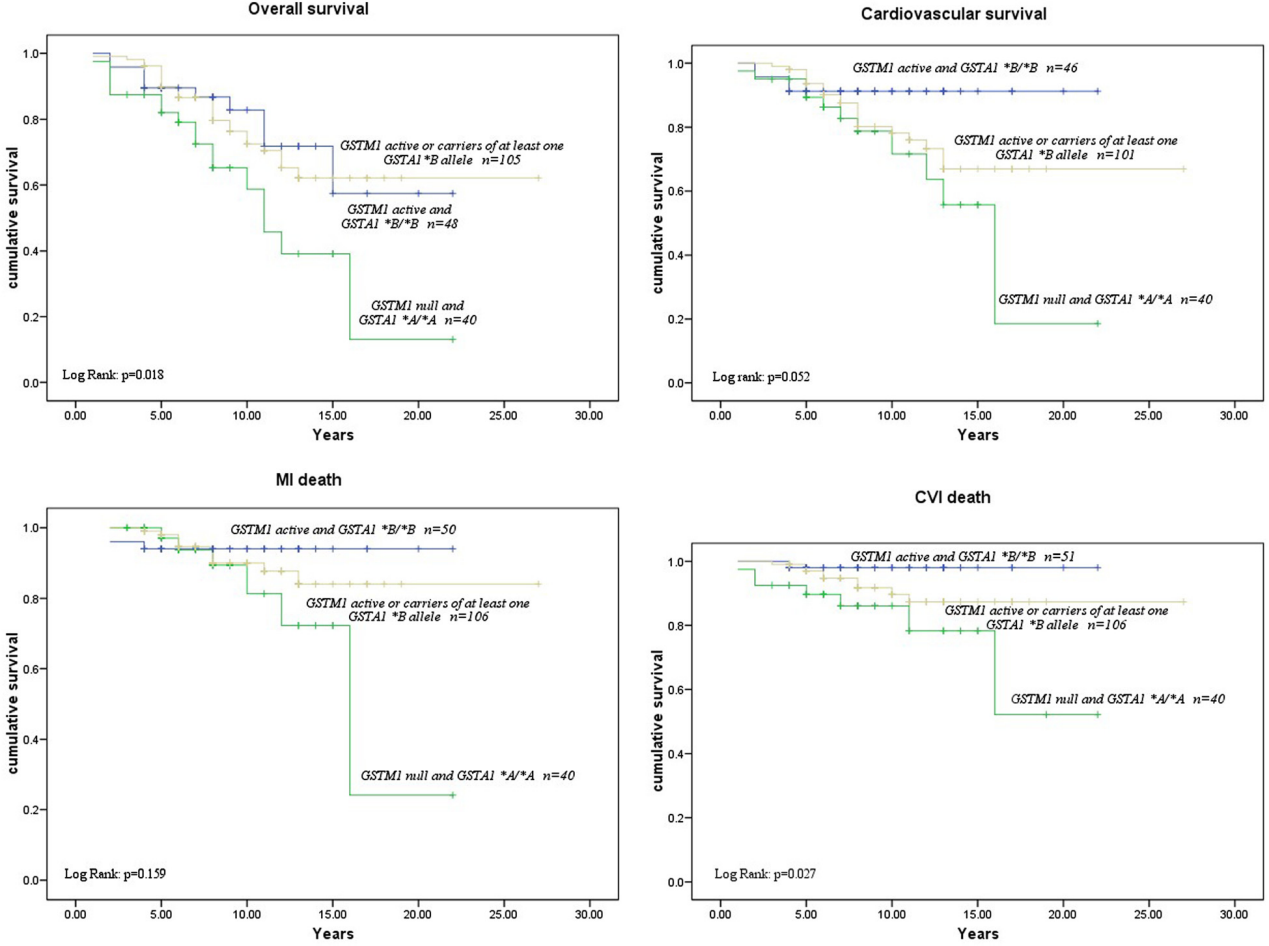
**Kaplan Meier Survival Curves for overall and cardiovascular mortality as well as death of myocardial infarction (MI) and stroke according to combined effect of *GSTM1/GSTA1 *** **polymorphisms.**

Interactive effects between GSTM1*0/0 and the GSTA1*A/A genotypes on the risk of various outcomes in ESRD patients are presented in Table 4. Regarding the risk for overall or cardiovascular mortality Cox regression analysis did not show interactive effects between *GSTM1* and *GSTA1* polymorphisms. However, patients with combined *GSTM1-null* and GSTA1*A/*A genotype exhibited a higher, although, non-significant multivariable adjusted (Model 3) HR of 2.27 (95% CI:0.81-6.38, P = 0.121) for MI, compared to individual polymorphisms (Table 4). Moreover, statistically significant interactive effect existed between *GSTM1-null* and the GSTA1*A/*A genotype for death of stroke. Specifically, dialysis patients who were homozygous for both GSTM1*0 and GSTA1*A alleles exhibited a HR of 4.38 (95%CI:1.50-12.75, P = 0.007) for stroke in comparison with carriers of at least one *GSTM1-active* and/or GSTA1*B allele. However after multiple testing (Additional file 1: Tables S3A and S3B and Additional file 4: Table S4) none of models remained significant. Additionally, in controlling for multiple testing, we excluded tests for GSTM1 and GSTA1 genes as they are marginal to GSTM1/GSTA1 interaction. A Kaplan-Meier survival analysis showed a significant association of combined *GSTM1* and *GSTA1* polymorphisms on ESRD patients survival (Figure 3). Namely, this analysis demonstrated a significantly shorter overall (Log Rank: p = 0.018) and cardiovascular survival (Log Rank: p = 0.052) as well as shorter time to death of MI (Log Rank: p = 0.159) or stroke (Log Rank: p = 0.027) after the initiation of dialysis in patients with combinedGSTM1*0/0 and GSTA1*A/A genotype in comparison with carriers of at least one *GSTM1-active* or GSTA1*B allele (Figure 3). Patients with combined *GSTM1-active* and GSTA1*B/B genotype had the best survival rate (Figure 3).

**Table 4:** Combined effect of GSTM1/GSTA1 polymorphisms as predictors for overall and cardiovascular mortality as well as death of myocardial infarction and cerebral vascular insult among 199 ESRD patients after a median follow-up time of 8 yrs by Cox proportional hazards regression models

**Model 1**^**a**^		**Model 2**^**b**^		**Model 3**^**c**^	
**HR (95% CI)**	**P value**	**HR (95% CI)**	**P value**	**HR (95% CI)**	**P value**
Risk for overall mortality comparing combined GSTM1*0/0 and GSTA1*A/A homozygotes to carriers of at least one *GSTM1*-active and/or GSTA1*B **allele**					
1.86 (1.03-3.36)	0.039	1.98 (1.10-3.58)	0.023	2.08 (1.13-3.83)	0.019
Risk for cardiovascular mortality comparing combined GSTM1*0/0 and GSTA1*A/A **homozygotes to carriers of at least one *GSTM1-*** active and/or GSTA1*B **allele**					
1.88 (0.93-3.82)	0.078	1.92 (0.95-3.91)	0.070	2.06 (0.98-4.34)	0.057
Risk for death of MI comparing combined GSTM1*0/0 and GSTA1*A/A **homozygotes to carriers of at least one *GSTM1-*** active and/or GSTA1*B **allele**					
2.23 (0.86-5.80)	0.099	2.25 (0.86-5.89)	0.098	2.27 (0.81-6.38)	0.121
Risk for death of CVI comparing combined GSTM1*0/0 and GSTA1*A/A **homozygotes to carriers of at least one *GSTM1 *** active and/or GSTA1*B **allele**					
3.35 (1.22-9.21)	0.019	3.55 (1.28-9.84)	0.015	4.38 (1.50-12.75)	0.007

*Abbreviations*: *CI* Confidence Interval, *HR *Hazard Ratio, *CVI *Cerebral Vascular Insult, *MI* Myocardial Infarction.

^a^Adjusted for age and gender.

^b^Adjusted for the covariates in Model 1 plus an additional adjustment for smoking status.

^c^Adjusted for the covariates in Model 2 plus an additional adjustment for diabetes and cholesterol level.

## Discussion

The data obtained in this study have shown that *GSTM1-null* genotype in ESRD patients is a significant independent predictor of overall and cardiovascular mortality. Besides, homozygous carriers of wild type GSTA1*A allele exhibited an increased risk of cardiovascular death, specifically MI and stroke, although the observed HR did not reach statistical significance in various models tested. The significant effect modification by GSTA1*A/A genotype on associations between *GSTM1-null* and analyzed outcomes was found only for the death from stroke. Homozygous carriers of combined GSTM1*0/GSTA1*A genotype exhibited significantly shorter time to MI or stroke in comparison to ESRD patients carriers of either *GSTM1-active* or GSTA1*B gene variant. The best survival rate in terms of cardiovascular outcome was found for ESRD patients with combined *GSTM1-active* and mutant GSTA1*B/B genotype.

Several lines of evidence indicate an association between the GSTM1*0/0 genotype and faster progression of kidney disease as well as worse outcome of dialysis patients. Very recently Chang et al. [21] have shown that *GSTM1-null* allele influences the course of kidney disease progression in participants of the African American Study of Kidney Disease (AASK) trial. Patient groups with and without the common *GSTM1-null* allele, differed significantly in the time to a glomerular filtration rate event or dialysis, or death. Similarly, Lin et al. [8] showed that dialysis patients with the GSTM1*0/0 genotype exhibit higher mortality rate when compared to those with the active enzyme. Our results confirmed these findings regarding the deleterious effect of GSTM1*0/0 genotype on overall mortality in ESRD patients. Furthermore, by the cause-specific analysis of the association between *GSTM1* and cardiovascular causes of death, such as MI and stroke, we provided a direct proof of the role of GSTM1 protein in prevention of oxidative stress related cardiovascular complications. Namely, patients lacking GSTM1 protein (GSTM1*0/0 genotype) demonstrated a significantly higher risk of cardiovascular death. When ESRD patients were further stratified according to the specific cause of death (MI or stroke), Kaplan Meier analysis demonstrated a significantly shorter time to death from both cardiovascular causes in patients with GSTM1*0/0 genotype in comparison to those with the active enzyme. The independent significant association between GSTM1*0/0 genotype remained only for the death from stroke as shown by Cox regression analysis. To our knowledge this is the first report showing a significant association between *GSTM1-null* genotype and the death from stroke in ESRD patients. These results are biologically plausible given the role of *GSTM1* in antioxidant protection and the progression of carotid atherosclerosis in pro-oxidant environment. Oxidized lipids are GST substrates while *GSTM1* directly regulates intracellular levels of lipid peroxidation by product 4-hydroxynonenal (4-HNE) in vascular smooth muscle cells [18]. A prospective study in Netherlands confirmed an increased progression of atherosclerosis among smokers lacking the enzyme GSTM1 [22]. Specifically, de Waart et al. demonstrated that the male smokers with the *GSTM1* genotype had a higher mean 2-yr progression of the common carotid artery intima-media thickness compared to those with *GSTM1*[22]. Although in this study we did not address the progression of carotid atherosclerosis in relation to *GSTM1* genotype, it may be speculated that the deleterious effect of *GSTM1-null* genotype might be even more pronounced in ESRD patients than in smokers, because of their higher exposure to dialysis-related oxidant stress. Having in mind that in chronic kidney disease the GSTM1 is normally up-regulated in a protective response to increased oxidative stress, as well as that the GSTM1 could be a surrogate for unmeasured oxidative stress markers [17,23], it seems reasonable to assume that this genetic variant may be deleterious in terms of carotid atherosclerosis and consequent stroke in ESRD patients.

The question arises about the association between *GSTM1-null* genotype and death from myocardial infarction being weaker than that observed for stroke. A recent meta-analysis suggested that the GSTM1*0/0 genotype associated with an increased risk of ischemic heart disease (OR, 1.38; 95% CI, 1.01 to 1.87) [24,25]. Although the presence of double deletion genotypes of the *GSTM1* gene is associated with hypertriglyceridemia and low HDL-cholesterol levels in healthy humans [26], it has not been shown directly that the null alleles actually change cardiac or coronary vascular GST activity. Assuming a change in activity, we cannot exclude a compensatory increase in expression of other *GSTs* in myocardium of *M1-null* genotypes in condition of dialysis related oxidative stress. Unlike humans, mice and rats do not possess the *GST-null* genotypes. However, genetic variation in expression of *Gstm1* in mice has been associated with differences in vascular smooth muscle cell proliferation, reactive oxygen species production, and cell migration in one study [27]. Updated meta-analyses of risk of IHD as a function of GSTM1*0/0 and GSTT1*0/0 genotypes in all studies combined, did not show an association of GST*0/0 genotype with the risk of ischemic heart disease, with the exception of *GSTM1*[28]. These results are in line with the lack of association of double deletion in *GSTT1* genotype and cardiovascular death risk in our cohort of ESRD patients.

Interestingly, in this study we found effect modification for the association between GSTM1*0/0 genotype and cardiovascular mortality by GSTA1*A/A genotype, which is associated with higher transcriptional activity and consequent higher levels of GSTA1 enzyme. This result was unexpected since in several non-malignant diseases GSTA1*B allele with lower transcriptional activity was associated with increased risk. Thus in Japanese, GSTA1*B allele is a potential risk factor for smoking-related type 2 diabetes and hypertension [29]. The interpretation of our results on effect modification by GSTA1*A/A genotype in GSTM1*0/0 individuals regarding the death from MI and stroke should be in the light of the fact that ESRD patients exhibit powerful oxidant stress, and therefore a strong Nrf mediated induction of GST expression [30]. GSTA1 protein belongs to the most promiscuous GSTs that acts upon a broad range of substrates which bind to its active site [31-33]. However, conjugation with GSH by means of GST enzymes sometimes results in the formation of more reactive compounds [34-36]. It may be speculated that some of the accumulated uremic toxins is converted into more reactive intermediates by GSTA1 protein. Although such an assumption still has to be documented, ESRD is not the only a disease in which GSTA1*A allele confers an increased risk. Namely, homozygous wild-type *GSTA1* genotype is associated with an increased risk of gastric cancer in Vietnamese patients [37].

Taken together, our data suggest that GSTM1*0/0 genotype and GSTM1*0/GSTA1*A genotype are the risk factors for cardiovascular death in ESRD patients, although after multiple testing our models missed statistical significance. These genetic markers may permit the targeting of preventive and early intervention on high-risk patients to reduce their cardiovascular risk. Specifically, studies are needed to reliably assess the effects of antioxidant therapy in people with GSTM1*0/0 genotype. In accordance with our previous findings these data suggest that the patients with GSTM1*0/0 genotype represent potential candidates for antioxidant therapy with the aim of stroke prevention. Although the antioxidant therapy does not reduce the risk of cardiovascular and all-cause death or major cardiovascular events in people with chronic kidney disease, according to the latest systematic review and metaanalysis [38,39] it is possible that some patients with particular genetic milieu may have a benefit from such therapy.

Certain limitations could be considered in our study. Relatively small numbers of both study participants and *GST* polymorphisms studied might be sources of potential biases which may influence the study findings. Formal power calculation revealed a 1-beta = 0.68. However, we tested effects of four *GST* polymorphisms on different treatment outcomes in 199 ESRD patients and therefore significantly decreased a chance for publication bias. Namely, positive results of genetic studies analyzing a small number of polymorphisms (n = 1-3) should be evaluated cautiously and considered at a lower level of evidence [40]. Another important point in our study is the low number of events (55 deaths, 36 of which due to cardiovascular causes) which may influence definitive conclusions about relationships between the polymorphisms and some relevant clinical outcomes. Additionally, after multiple testing none of models remained significant. Besides that, survival bias could be present due to the fact that an increased hazard for cardiovascular events in the chronic kidney disease patients may contribute to the death of these persons prior to reaching dialysis and consequently to the under-representation of the polymorphism effects in our cohort. Therefore the results of our study require validation in other dialysis cohorts.

## Conclusion

In conclusion, combined GSTM1*0/GSTA1*A genotypes might be considered as genetic markers for cardiovascular death risk in ESRD patients, which may permit targeting of preventive and early intervention. Based on our results, it can be assumed that patients with particular genetic milieu might have a benefit from antioxidant therapy.

## Competing interests

The author(s) declare that they have no competing interests, including specific financial interests and relationships and affiliations relevant to the subject of this manuscript.

## Authors’ contributions

SS was responsible for patients’ recruitment, conducting laboratory experimental procedures and their interpretation and writing of the manuscript. TS was responsible for the design of the study and manuscript writing. TP and JJ were responsible for statistical analysis and revision of the manuscript. TD, SP and ND were responsible for patients recruitment, clinical diagnosis and participated in scientific discussions and revision of the manuscript. ASR, MPE, JMO, SR and DS have made substantial contributions to analysis and interpretation of data and revision of the manuscript. MZ was responsible for multiple testing analysis. All authors read and approved the final manuscript.

## Pre-publication history

The pre-publication history for this paper can be accessed here:

http://www.biomedcentral.com/1471-2369/15/12/prepub

## Supplementary Material

Additional file 1: Table S3 A. Multiplicity correction and false discovery rate estimation for GSTA1, GSTM1, GSTP1 and GSTT1 polymorphisms as a predictors of different outcomes among 199 ESRD patients. B. Multiplicity correction and false discovery rate estimation for combined GSTA1/GSTM1, GSTP1 and GSTT1 polymorphisms as a predictors of different outcomes among 199 ESRD patients. Click here for file

Additional file 2: Table S1 *GSTT1* polymorphism as a predictor for overall and cardiovascular mortality as well as death of myocardial infarction and cerebral vascular insult among 199 ESRD patients after a median follow-up time of 8 yrs by Cox proportional hazards regression models. Click here for file

Additional file 3: Table S2 GSTP1 polymorphism as a predictor for overall and cardiovascular mortality as well as death of myocardial infarction and cerebral vascular insult among 199 ESRD patients after a median follow-up time of 8 yrs by Cox proportional hazards regression models. Click here for file

Additional file 4: Table S4 Urea and creatinine in ERSD patients according to different GSTA1, GSTM1, GSTP1 and GSTT1 genotypes. Click here for file
