# Supplementary material for: Associations of GSTM1*0 and GSTA1*A genotypes with the risk of cardiovascular death among hemodialyses patients
Source: BMC Nephrol. 2014 Jan 14;15:12. doi: 10.1186/1471-2369-15-12 (PMC3909531; doi:10.1186/1471-2369-15-12)
Supplement: Additional file 1: Table S3 — A. Multiplicity correction and false discovery rate estimation for GSTA1, GSTM1, GSTP1 and GSTT1 polymorphisms as a predictors of different outcomes among 199 ESRD patients. B. Multiplicity correction and false discovery rate estimation for combined GSTA1/GSTM1, GSTP1 and GSTT1 polymorphisms as a predictors of different outcomes among 199 ESRD patients. [file 1471-2369-15-12-S1.docx]

**Additional file 1: Table S3A**. Multiplicity correction and false discovery rate estimation for GSTA1, GSTM1, GSTP1 and GSTT1 polymorphisms as a predictors of different outcomes among 199 ESRD patients

|  |  | Model 1 | | | | | Model 2 | | | | | Model 3 | | | | |
| --- | --- | --- | --- | --- | --- | --- | --- | --- | --- | --- | --- | --- | --- | --- | --- | --- |
|  | Outcome |  | FWER | | FDR | |  | FWER | | FDR | |  | FWER | | FDR | |
|  |  | raw | Bonferroni^a^ | Hochberg^b^ | BH^c^ | TSB^d^ | raw | Bonferroni^a^ | Hochberg^b^ | BH^c^ | TSB^d^ | raw | Bonferroni^a^ | Hochberg^b^ | BH^c^ | TSB^d^ |
| GSTM1 | Overall mortality | 0.07 | 1.00 | 0.78 | 0.26 | 0.26 | 0.04 | 0.82 | 0.74 | 0.23 | 0.23 | 0.02 | 0.48 | 0.43 | 0.16 | 0.16 |
|  | CVM | 0.07 | 1.00 | 0.78 | 0.26 | 0.26 | 0.06 | 1.00 | 0.83 | 0.23 | 0.23 | 0.05 | 1.00 | 0.80 | 0.19 | 0.19 |
|  | MI | 0.28 | 1.00 | 0.78 | 0.51 | 0.51 | 0.26 | 1.00 | 0.83 | 0.48 | 0.48 | 0.27 | 1.00 | 0.89 | 0.49 | 0.49 |
|  | CVI | 0.06 | 1.00 | 0.78 | 0.26 | 0.26 | 0.05 | 1.00 | 0.83 | 0.23 | 0.23 | 0.05 | 0.90 | 0.77 | 0.19 | 0.19 |
| GSTA1 | overall mortality | 0.56 | 1.00 | 0.78 | 0.70 | 0.70 | 0.64 | 1.00 | 0.83 | 0.82 | 0.82 | 0.65 | 1.00 | 0.89 | 0.86 | 0.86 |
|  | CVM | 0.16 | 1.00 | 0.78 | 0.36 | 0.36 | 0.18 | 1.00 | 0.83 | 0.39 | 0.39 | 0.13 | 1.00 | 0.89 | 0.30 | 0.30 |
|  | MI | 0.26 | 1.00 | 0.78 | 0.51 | 0.51 | 0.26 | 1.00 | 0.83 | 0.48 | 0.48 | 0.23 | 1.00 | 0.89 | 0.46 | 0.46 |
|  | CVI | 0.12 | 1.00 | 0.78 | 0.30 | 0.30 | 0.11 | 1.00 | 0.83 | 0.27 | 0.27 | 0.09 | 1.00 | 0.89 | 0.26 | 0.26 |
| GSTM1/GSTA1 | overall mortality | 0.04 | 0.78 | 0.74 | 0.26 | 0.26 | 0.02 | 0.46 | 0.44 | 0.23 | 0.23 | 0.02 | 0.38 | 0.36 | 0.16 | 0.16 |
|  | CVM | 0.08 | 1.00 | 0.78 | 0.26 | 0.26 | 0.07 | 1.00 | 0.83 | 0.23 | 0.23 | 0.06 | 1.00 | 0.86 | 0.19 | 0.19 |
|  | MI | 0.10 | 1.00 | 0.78 | 0.28 | 0.28 | 0.10 | 1.00 | 0.83 | 0.27 | 0.27 | 0.12 | 1.00 | 0.89 | 0.30 | 0.30 |
|  | CVI | 0.02 | 0.38 | 0.38 | 0.26 | 0.26 | 0.02 | 0.30 | 0.30 | 0.23 | 0.23 | 0.01 | 0.14 | 0.14 | 0.14 | 0.14 |
| GSTT1 | overall mortality | 0.71 | 1.00 | 0.78 | 0.75 | 0.75 | 0.76 | 1.00 | 0.83 | 0.82 | 0.82 | 0.89 | 1.00 | 0.89 | 0.89 | 0.89 |
|  | CVM | 0.67 | 1.00 | 0.78 | 0.74 | 0.74 | 0.73 | 1.00 | 0.83 | 0.82 | 0.82 | 0.88 | 1.00 | 0.89 | 0.89 | 0.89 |
|  | MI | 0.46 | 1.00 | 0.78 | 0.70 | 0.70 | 0.51 | 1.00 | 0.83 | 0.77 | 0.77 | 0.57 | 1.00 | 0.89 | 0.86 | 0.86 |
|  | CVI | 0.78 | 1.00 | 0.78 | 0.78 | 0.78 | 0.83 | 1.00 | 0.83 | 0.83 | 0.83 | 0.87 | 1.00 | 0.89 | 0.89 | 0.89 |
| GSTP1 | overall mortality | 0.56 | 1.00 | 0.78 | 0.70 | 0.70 | 0.52 | 1.00 | 0.83 | 0.77 | 0.77 | 0.64 | 1.00 | 0.89 | 0.86 | 0.86 |
|  | CVM | 0.60 | 1.00 | 0.78 | 0.70 | 0.70 | 0.78 | 1.00 | 0.83 | 0.82 | 0.82 | 0.83 | 1.00 | 0.89 | 0.89 | 0.89 |
|  | MI | 0.41 | 1.00 | 0.78 | 0.68 | 0.68 | 0.54 | 1.00 | 0.83 | 0.77 | 0.77 | 0.69 | 1.00 | 0.89 | 0.86 | 0.86 |
|  | CVI | 0.57 | 1.00 | 0.78 | 0.70 | 0.70 | 0.68 | 1.00 | 0.83 | 0.82 | 0.82 | 0.40 | 1.00 | 0.89 | 0.67 | 0.67 |

CVM- cardiovascular mortality; MI- myocardial infarction; CVI- cerebral vascular insult; FWER family-wise error rate; FDR false discovery rate;

^a^Bonferroni corrected p;

^b^ Hochberg corrected p

^c^BH Adjusted p-values for the Benjamini & Hochberg (1995) FDR-controlling procedure

^d^TSB Adjusted p-values for the Two-stage Benjamini, Krieger & Yekutieli (2006) FDR procedure

**Additional file 1: Table S3B**. Multiplicity correction and false discovery rate estimation for combined GSTA1/GSTM1, GSTP1 and GSTT1 polymorphisms as a predictors of different outcomes among 199 ESRD patients

|  |  | Model 1 | | | | | Model 2 | | | | | Model 3 | | | | |
| --- | --- | --- | --- | --- | --- | --- | --- | --- | --- | --- | --- | --- | --- | --- | --- | --- |
|  | outcome |  | FWER | | FDR | |  | FWER | | FDR | |  | FWER | | FDR | |
|  |  | raw | Bonferroni^a^ | Hochberg^b^ | BH^c^ | TSB^d^ | raw | Bonferroni^a^ | Hochberg^b^ | BH^c^ | TSB^d^ | raw | Bonferroni^a^ | Hochberg^b^ | BH^c^ | TSB^d^ |
| GSTM1/GSTA1 | Overall mortality | 0.04 | 0.47 | 0.43 | 0.23 | 0.23 | 0.02 | 0.28 | 0.25 | 0.14 | 0.14 | 0.02 | 0.23 | 0.21 | 0.11 | 0.11 |
|  | CVM | 0.08 | 0.94 | 0.78 | 0.30 | 0.30 | 0.07 | 0.84 | 0.70 | 0.28 | 0.28 | 0.06 | 0.68 | 0.57 | 0.23 | 0.23 |
|  | MI | 0.10 | 1.00 | 0.78 | 0.30 | 0.30 | 0.10 | 1.00 | 0.83 | 0.29 | 0.29 | 0.12 | 1.00 | 0.89 | 0.36 | 0.36 |
|  | CVI | 0.02 | 0.23 | 0.23 | 0.23 | 0.23 | 0.02 | 0.18 | 0.18 | 0.14 | 0.14 | 0.01 | 0.08 | 0.08 | 0.08 | 0.08 |
| GSTT1 | Overall mortality | 0.71 | 1.00 | 0.78 | 0.78 | 0.78 | 0.76 | 1.00 | 0.83 | 0.83 | 0.83 | 0.89 | 1.00 | 0.89 | 0.89 | 0.89 |
|  | CVM | 0.67 | 1.00 | 0.78 | 0.78 | 0.78 | 0.73 | 1.00 | 0.83 | 0.83 | 0.83 | 0.88 | 1.00 | 0.89 | 0.89 | 0.89 |
|  | MI | 0.46 | 1.00 | 0.78 | 0.78 | 0.78 | 0.51 | 1.00 | 0.83 | 0.83 | 0.83 | 0.57 | 1.00 | 0.89 | 0.89 | 0.89 |
|  | CVI | 0.78 | 1.00 | 0.78 | 0.78 | 0.78 | 0.83 | 1.00 | 0.83 | 0.83 | 0.83 | 0.87 | 1.00 | 0.89 | 0.89 | 0.89 |
| GSTP1 | Overall mortality | 0.56 | 1.00 | 0.78 | 0.78 | 0.78 | 0.52 | 1.00 | 0.83 | 0.83 | 0.83 | 0.64 | 1.00 | 0.89 | 0.89 | 0.89 |
|  | CVM | 0.60 | 1.00 | 0.78 | 0.78 | 0.78 | 0.78 | 1.00 | 0.83 | 0.83 | 0.83 | 0.83 | 1.00 | 0.89 | 0.89 | 0.89 |
|  | MI | 0.41 | 1.00 | 0.78 | 0.78 | 0.78 | 0.54 | 1.00 | 0.83 | 0.83 | 0.83 | 0.69 | 1.00 | 0.89 | 0.89 | 0.89 |
|  | CVI | 0.57 | 1.00 | 0.78 | 0.78 | 0.78 | 0.68 | 1.00 | 0.83 | 0.83 | 0.83 | 0.40 | 1.00 | 0.89 | 0.89 | 0.89 |

CVM- cardiovascular mortality; MI- myocardial infarction; CVI- cerebral vascular insult; FWER family-wise error rate; FDR false discovery rate;

^a^Bonferroni corrected p;

^b^ Hochberg corrected p

^c^BH Adjusted p-values for the Benjamini & Hochberg (1995) FDR-controlling procedure

^d^TSB Adjusted p-values for the Two-stage Benjamini, Krieger & Yekutieli (2006) FDR procedure
