# Supplementary material for: Associations of GSTM1*0 and GSTA1*A genotypes with the risk of cardiovascular death among hemodialyses patients
Source: BMC Nephrol. 2014 Jan 14;15:12. doi: 10.1186/1471-2369-15-12 (PMC3909531; doi:10.1186/1471-2369-15-12)
Supplement: Additional file 2: Table S1 — GSTT1 polymorphism as a predictor for overall and cardiovascular mortality as well as death of myocardial infarction and cerebral vascular insult among 199 ESRD patients after a median follow-up time of 8 yrs by Cox proportional hazards regression models. [file 1471-2369-15-12-S2.doc]

Additional file 2: Table S1.*GSTT1* polymorphism as a predictor for overall and cardiovascular mortality as well as death of myocardial infarction and cerebral vascular insult among 199 ESRD patients after a median follow-up time of 8 yrs by Cox proportional hazards regression models

| Model 1^a^ | | Model 2^b^ | | Model 3^c^ | |
| --- | --- | --- | --- | --- | --- |
| HR (95% CI) | P value | HR (95% CI) | P value | HR (95% CI) | P value |
| **Risk for overall mortality comparing *GSTT1-null* homozygotes to *GSTT1-active* carriers** | | | | | |
| 0.89 (0.50-1.62) | 0.713 | 0.91 (0.50-1.65) | 0.756 | 0.96 (0.51-1.80) | 0.892 |
| **Risk for cardiovascular mortality comparing *GSTT1-null* homozygotes to *GSTT1-active* carriers** | | | | | |
| 0.86 (0.42-1.74) | 0.668 | 0.88 (0.43-1.81) | 0.733 | 0.94 (0.44-2.03) | 0.875 |
| **Risk for death from myocardial infarction comparing *GSTT1-null* homozygotes to *GSTT- active* carriers** | | | | | |
| 0.68 (0.24-1.90) | 0.463 | 0.71 (0.25-2.00) | 0.514 | 0.71 (0.23-2.26) | 0.566 |
| **Risk for death from CVI comparing *GSTT- null* homozygotes to *GSTT1-active* carriers** | | | | | |
| 0.86 (0.30-2.49) | 0.779 | 0.89 (0.30-2.59) | 0.825 | 1.10 (0.36-3.39) | 0.865 |

Abbreviations: CI, Confidence Interval; HR, Hazard Ratio.

^a^Adjusted for age and gender.

^b^Adjusted for the covariates in Model 1 plus an additional adjustment for smoking status.

^c^Adjusted for the covariates in Model 2 plus an additional adjustment for diabetes and cholesterol level.
