# Supplementary material for: Associations of GSTM1*0 and GSTA1*A genotypes with the risk of cardiovascular death among hemodialyses patients
Source: BMC Nephrol. 2014 Jan 14;15:12. doi: 10.1186/1471-2369-15-12 (PMC3909531; doi:10.1186/1471-2369-15-12)
Supplement: Additional file 3: Table S2 — GSTP1 polymorphism as a predictor for overall and cardiovascular mortality as well as death of myocardial infarction and cerebral vascular insult among 199 ESRD patients after a median follow-up time of 8 yrs by Cox proportional hazards regression models. [file 1471-2369-15-12-S3.doc]

Additional file 3: Table S2. GSTP1 polymorphism as a predictor for overall and cardiovascular mortality as well as death of myocardial infarction and cerebral vascular insult among 199 ESRD patients after a median follow-up time of 8 yrs by Cox proportional hazards regression models

| Model 1^a^ | | Model 2^b^ | | Model 3^c^ | |
| --- | --- | --- | --- | --- | --- |
| HR (95% CI) | P value | HR (95% CI) | P value | HR (95% CI) | P value |
| **Risk for overall mortality comparing *GSTP1 Val/Val* homozygotes to *GSTP1 Ile* carriers** | | | | | |
| 0.83 (0.45-1.55) | 0.561 | 0.81 (0.43-1.54) | 0.523 | 0.85 (0.42-1.71) | 0.643 |
| **Risk for cardiovascular mortality comparing *GSTP1 Val/Val* homozygotes to *GSTP1 Ile* carriers** | | | | | |
| 0.82 (0.39-1.72) | 0.597 | 0.90 (0.42-1.91) | 0.777 | 1.10 (0.46-2.60) | 0.833 |
| **Risk for death from myocardial infarction comparing *GSTP1 Val/Val* homozygotes to *GSTP1 Ile* carriers** | | | | | |
| 0.66 (0.25-1.76) | 0.406 | 0.73 (0.27-2.00) | 0.539 | 1.30 (0.36-4.74) | 0.690 |
| **Risk for death from CVI comparing *GSTP1 Val/Val* homozygotes to *GSTP1 Ile* carriers** | | | | | |
| 0.73 (0.25-2.15) | 0.572 | 0.80 (0.26-2.40) | 0.683 | 0.62 (0.20-1.90) | 0.402 |

Abbreviations: CI, Confidence Interval; HR, Hazard Ratio.

^a^Adjusted for age and gender.

^b^Adjusted for the covariates in Model 1 plus an additional adjustment for smoking status.

^c^Adjusted for the covariates in Model 2 plus an additional adjustment for diabetes and cholesterol level.
