# Supplementary material for: Associations of GSTM1*0 and GSTA1*A genotypes with the risk of cardiovascular death among hemodialyses patients
Source: BMC Nephrol. 2014 Jan 14;15:12. doi: 10.1186/1471-2369-15-12 (PMC3909531; doi:10.1186/1471-2369-15-12)
Supplement: Additional file 4: Table S4 — Urea and creatinine in ERSD patients according to different GSTA1, GSTM1, GSTP1 and GSTT1 genotypes. [file 1471-2369-15-12-S4.docx]

Additional file 4: Table S4. Urea and creatinine in ERSD patients according to different GSTA1, GSTM1, GSTP1 and GSTT1 genotypes

| Genotypes | | Urea (mmol/L) | Creatinine (μmol/L) |
| --- | --- | --- | --- |
| ***GSTA1^a^*** | A*/A* | 24.3±4.8 | 892±163 |
|  | A*/B* | 24.3±5.7 | 891±249 |
|  | B*/B* | 24.1±4.8 | 833±241 |
| ***GSTM1*** | Active | 24.3±5.3 | 873±250 |
|  | Null | 24.2±5.1 | 868±227 |
| ***GSTP1*** | Ile/Ile | 24.4±4.9 | 912±230 |
|  | Ile/Val | 24.6±6.1 | 845±275 |
|  | Val/Val | 23.8±4.3 | 873±205 |
| ***GSTT1*** | Active | 23.9±5.1 | 868±278 |
|  | Null | 24.4±5.3 | 872±220 |
